# Use of renal contrast-enhanced ultrasound to explore renal cortical microcirculation abnormalities in pediatric acute kidney injury

**DOI:** 10.3389/fped.2025.1574398

**Published:** 2025-06-13

**Authors:** Chunbei Yi, Fang Zhang, Jing Shi, Jian Zhang, Jun Du

**Affiliations:** ^1^ Department of Diagnostic Imaging Center, Shanghai Children’s Medical Center, Shanghai Jiao Tong University School of Medicine, Shanghai, China; ^2^ Department of Pediatric Intensive Care Unit, Shanghai Children’s Medical Center, Shanghai Jiao Tong University School of Medicine, Shanghai, China

**Keywords:** acute kidney injury, contrast-enhanced ultrasound, renal perfusion, microcirculation, renal haemodynamics, child

## Abstract

**Objective:**

To explore the correlation between the quantitative analysis curve of renal contrast-enhanced ultrasound and the anatomical location of renal cortical microcirculation associated with acute kidney injury.

**Methods:**

This study included a 1-year-and-11-month-old female child with acute kidney injury caused by drug overdosed and a 14-year-old female child with acute kidney injury caused by drug intentional, who were treated at Shanghai Children's Medical Center affiliated with Shanghai Jiao Tong University School of Medicine. Both patients underwent renal contrast-enhanced ultrasound and quantitative analysis. In addition, their clinical medical history data were recorded.

**Results:**

The first child developed acute kidney injury owing to cyclosporine A overdosed. Contrast-enhanced ultrasound revealed poor cortical blood flow perfusion in both kidneys, with abnormally prolonged cortical perfusion times and possible obstruction of vascular inflow pathways. The second child experienced acute kidney injury owing to ibuprofen intentional. Contrast-enhanced ultrasound showed good cortical blood wash-in/perfusion but significantly delayed wash-out/excretion.

**Conclusion:**

The structure and function of the glomerulus significantly influence the perfusion rate and intensity of the rising branch of the curve. Furthermore, the descending branch of the curve is affected by the interplay of the capillaries surrounding the renal tubules. Exploration of these anatomical structures aids in understanding the renal microcirculation pathways and provides further insight into renal perfusion dynamics.

## Introduction

Acute kidney injury (AKI) is a clinical syndrome characterized by a rapid decline in renal function owing to various etiologies. While the pathogenesis of AKI differs depending on the causative factors, changes in renal blood flow perfusion are fundamental to its occurrence (1). The pathway of renal blood flow originates from the aorta, which gives rise to the left and right renal arteries. These arteries branch into smaller vessels within the kidney, extending to the interlobular arteries located in the renal cortex. The renal cortical blood supply comprises two primary capillary networks: (1) afferent arterioles → glomerular capillary network → efferent arterioles; and (2) peritubular capillary network → renal vein. The glomerular capillary network operates at higher blood pressure, facilitating glomerular filtration, while the peritubular capillary network functions at lower pressure, promoting tubular reabsorption. The urinary pathway begins with blood flowing through the glomerulus, where urine formation occurs through glomerular filtration and tubular reabsorption. The urine is then transported to the bladder via the ureters and finally expelled through the urethra. The patency of both the renal blood flow and urinary pathways reflects the functional state of the kidneys, with each pathway exerting a reciprocal influence on the other.

Contrast-enhanced ultrasound (CEUS) operates on the principle of visualizing the microvasculature by injecting ultrasound contrast agents into the bloodstream. This method facilitates both quantitative and qualitative assessments of renal parenchymal perfusion and allows for real-time visualization of renal anatomical structures (2). Additionally, the contrast microbubbles used in CEUS are excreted through the lungs, ensuring no renal toxicity (3).

Currently, there is a notable scarcity of research investigating the ascent and descent patterns of the quantitative analysis curve in renal CEUS and their correlation with the anatomical locations of renal microcirculation. This study aims to investigate this relationship and assess its clinical significance.

## Methods

### Research objective

Patients who developed AKI during hospitalization in the Department of Pediatric Intensive Care Unit of Shanghai Children's Medical Center, Shanghai Jiao Tong University School of Medicine, from September 2023 to May 2024 were recruited. Patients who met the diagnostic criteria for AKI caused by drug overdose or intentional and those aged ≤18 years were included. Those with the presence of a right-to-left cardiac shunt, severe pulmonary hypertension, or poorly controlled hypertension were excluded from the study.

### Ultrasound examination and quantitative analysis

A Resonna R9 and a portable ultrasound M11 (Mindray, Shen Zhen, China) equipped with a convex array probe (frequency: 6 MHz) were used in this study. Conventional ultrasound and color Doppler imaging were performed before CEUS. In CEUS mode, imaging was conducted at the maximum longitudinal section of the kidney. A bolus dose of 0.03 ml/kg of microbubbles containing sulfur hexafluoride (SonoVue, Bracco, Italy) was injected intravenously into an antecubital or central vein, followed by a flush with 5 ml of normal saline. Real-time dynamic CEUS images were recorded concurrently. After a 15-min interval, the same procedure was repeated for the contralateral kidney. Quantitative analysis was performed using the built-in software of the ultrasound machine. The region of interest was placed perpendicular to the renal cortex of the ultrasound beam, carefully avoiding the thicker blood vessels. The software automatically generated a time-intensity curve (TIC) and provided CEUS parameters, including peak intensity, rise time, area under the curve, and time to peak, among others.

## Results

### Clinical data

A 1-year-and-11-month-old child with a confirmed diagnosis of mucopolysaccharidosis type I developed AKI owing to cyclosporine A (CsA) overdose after a second hematopoietic stem cell transplant. Renal function tests on admission showed elevated levels of urea at 9.4 mmol/L, creatinine at 34 µmol/L, and uric acid at 407.2 µmol/L. Blood pressure fluctuated considerably, with systolic blood pressure ranging from 180 to 200 mmHg and diastolic pressure ranging from 60 to 100 mmHg during periods of restlessness, stabilizing to 120–140/50–70 mmHg at rest. CEUS indicated poor cortical blood flow perfusion in both kidneys, with abnormally prolonged cortical perfusion times and possible obstruction of vascular inflow pathways.

The other patient, a 14-year-old child, had accidentally ingested a large quantity of ibuprofen (108 sustained-release tablets, each containing 0.4 g of ibuprofen) and developed AKI caused by nonsteroidal anti-inflammatory drug intentional. Renal function tests on admission showed a urea level of 19.0 mmol/L, creatinine level of 261 *μ*mol/L, uric acid level of 393.3 µmol/L, serum cystatin C level of 1.46 mg/L, and neutrophil gelatinase-associated lipocalin (NGAL) level of 267 ng/ml in serum and 442 ng/ml in urine. Electrolyte levels were as follows: K^+^: 5.0 mmol/L, Na^+^: 131 mmol/L, and Free Ca: 0.89 mmol/L. CEUS showed good cortical blood wash-in and perfusion but significantly slow wash-out and excretion.

### Analysis of US and CEUS parameters

**Figure 1:**
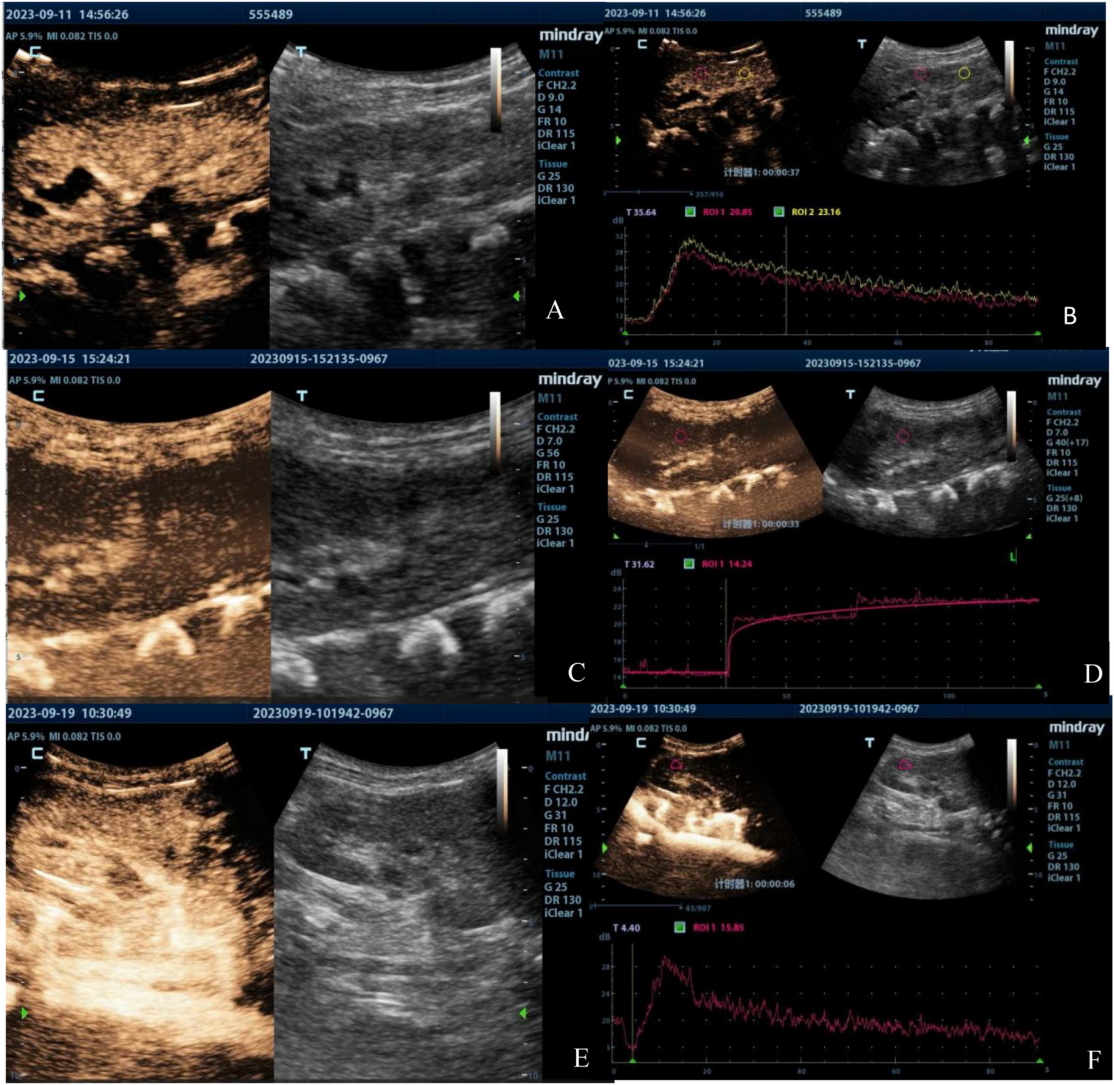
Contrast-enhanced ultrasound images of the right kidney of the first patient taken on Day 4 **(A,B)**, Day 8 **(C,D)**, and Day 12 **(E,F)**. The images illustrate the progression of renal perfusion changes over time, showing an initial reduction in perfusion, abnormal prolongation of perfusion time, and subsequent recovery by Day 12.

**Figure 2:**
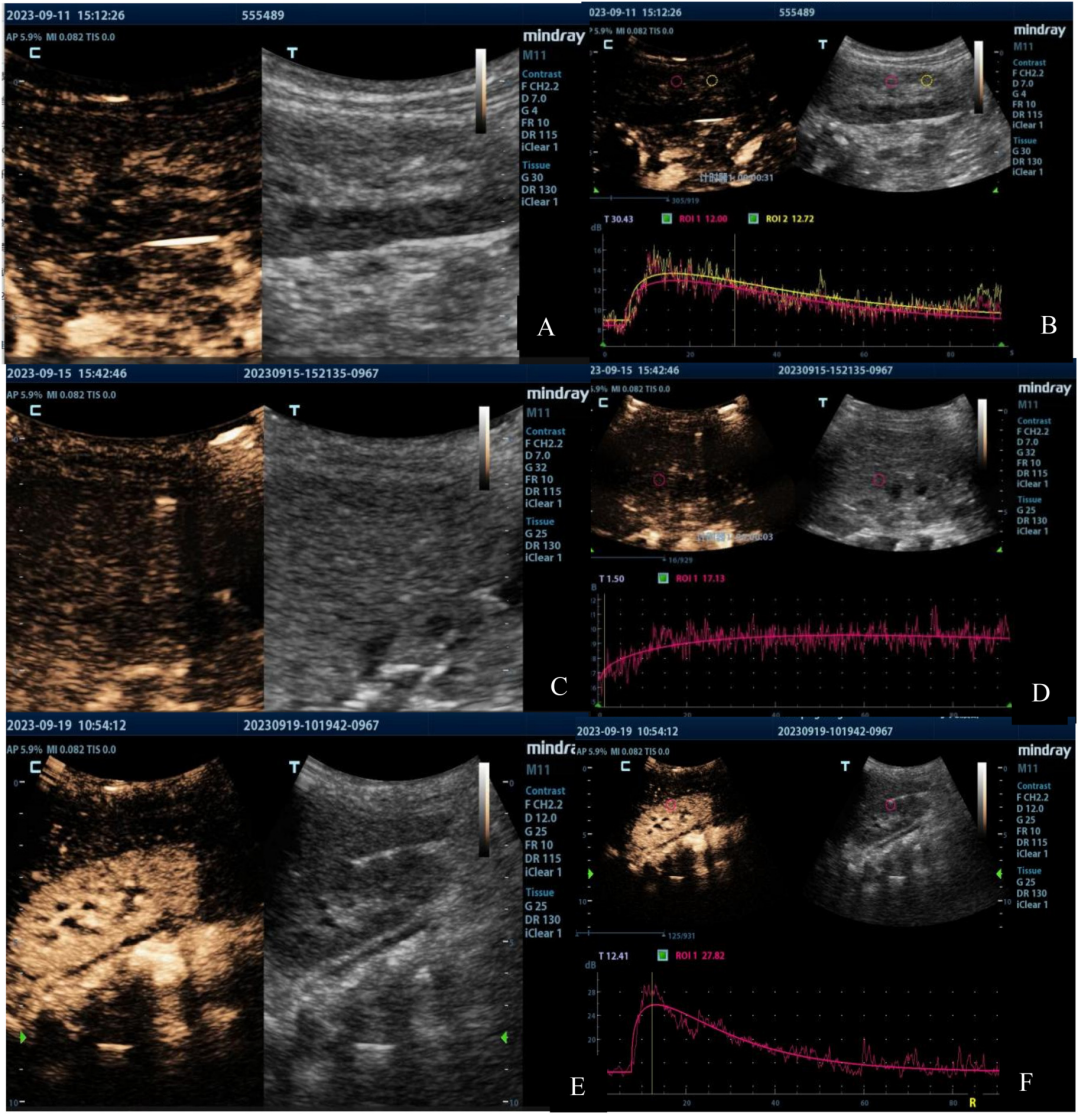
Contrast-enhanced ultrasound images of the left kidney in the first patient taken on Day 4 **(A,B)**, Day 8 **(C,D)**, and Day 12 **(E,F)**. The images demonstrate a significant decrease and abnormal prolongation of perfusion time, followed by marked improvement by Day 12.

Renal ultrasound results of the first patient indicated bilateral renal enlargement (right kidney: 81 mm × 33 mm, left kidney: 84 mm × 36 mm) and slightly elevated echoes in the parenchyma. Doppler ultrasound revealed sparse blood flow and increased resistive indexes in the interlobar arteries (right: 0.71, left: 0.77). CEUS on Day 4 of admission (90 s observation) showed that bilateral renal perfusion was slow and reduced, more prominently in the left kidney (Figures 1A, 2A). Despite reduced perfusion, excretion remained acceptable (Figures 1B, 2B). CEUS on Day 8 of admission (120 s observation) demonstrated that the main trunk of the right renal artery and intrarenal segmental arteries were progressively enhanced but with sparse visualization. Microbubbles infiltrated the renal parenchyma in a “scattered star” pattern, with significantly reduced enhancement (Figures 1C). The TIC exhibited a stepwise ascending pattern without any decline (Figures 1D). For the left renal artery, the main trunk and intrarenal segmental arteries were faintly and unclearly visualized, with microbubble enhancement in the renal parenchyma being poorly defined and extremely low, resulting in a flat, continuous upward TIC curve without any decline (Figures 2C,D).

The results of CEUS implied poor bilateral renal cortical blood flow perfusion, significantly prolonged bilateral renal cortical perfusion times, and a potential obstruction in the vascular inflow pathways. CEUS on Day 12 of admission (90 s observation) showed significant improvement in the perfusion of both kidneys compared to previous findings, with more noticeable improvement in the left kidney [Figures 1E,F, 2E,F]. Restoration of arterial inflow pathways appeared to be progressing in both kidneys. Quantitative parameters were presented at Table 1.

**Table 1:** Quantitative parameters of CEUS in first patient.

Midray M11	Date	TTP	RT	PI	AS	DS	AUC
Right	4d	20.80	15.50	15.14	0.75	0.17	1,811.39
	8d	127.90	93.60	8.16	0.04	NA	2,065.28
	12d	9.00	8.30	8.39	0.59	0.13	1,739.36
Left	4d	16.70	11.30	4.51	0.26	0.06	951.12
	8d	56.10	45.10	2.93	0.02	NA	1,769.78
	12d	15.30	7.80	15.13	0.95	0.21	1,757.69

CEUS, contrast-enhanced ultrasound; TTP, peak time in seconds; RT, rise time in seconds; PI, peak intensity in dB; AS, ascending slope; DS, descending slope; AUC, area under the curve.

**Figure 3:**
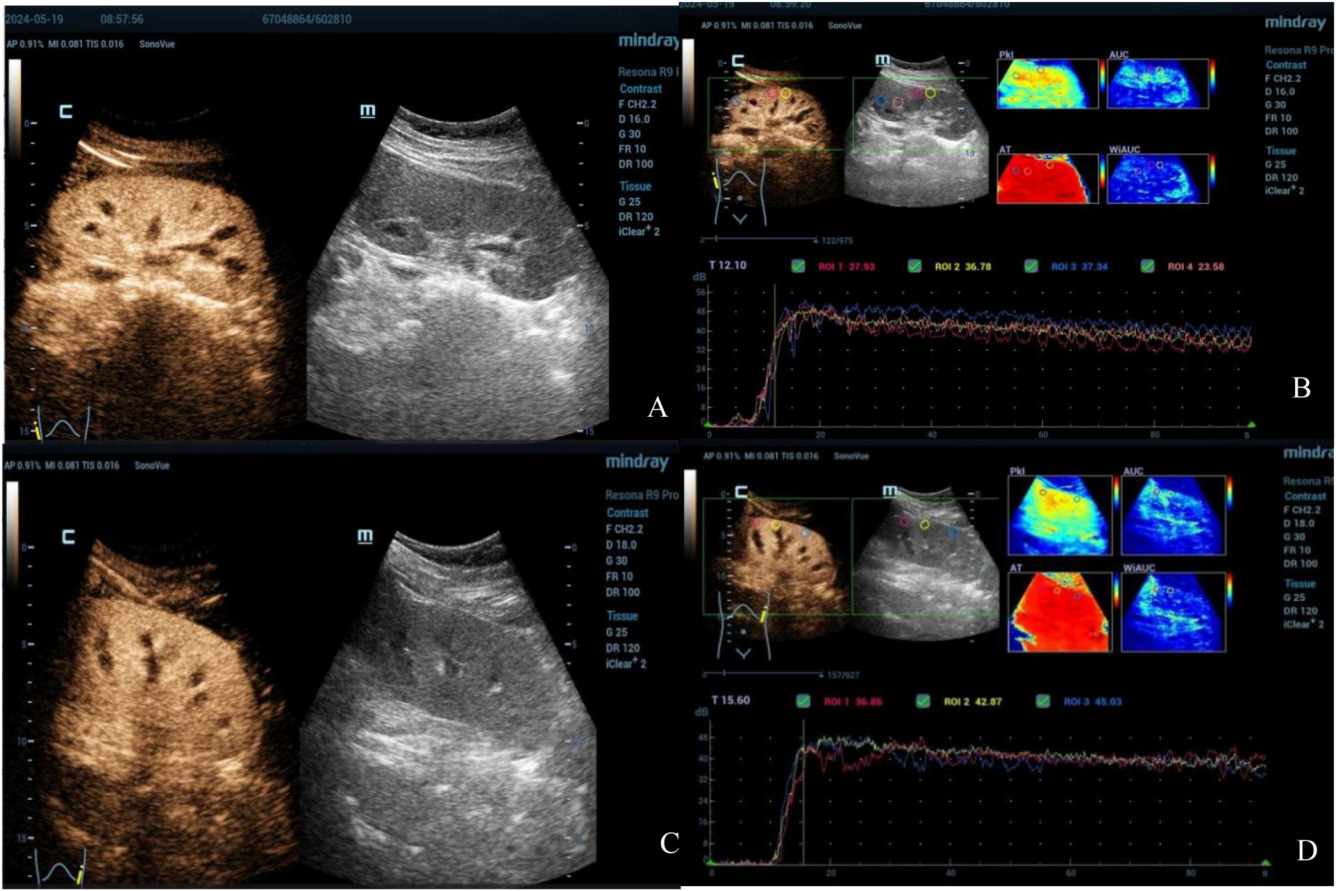
Contrast-enhanced ultrasound images of both kidneys in the second patient (Right: **A**,**B**; Left: **C**,**D**). The images reveal good cortical blood wash-in and perfusion but significantly delayed wash-out and excretion.

Renal ultrasound of the second patient also revealed bilateral renal enlargement (right kidney: 122 mm × 59 mm, left kidney: 127 mm × 60 mm) without abnormalities in echogenicity. Doppler ultrasound demonstrated good blood flow and relatively lower resistive indexes in the interlobar arteries (right: 0.59, left: 0.50). CEUS was conducted on Day 2 (90 s observation) when the patient's urine output had normalized and creatinine levels had decreased to 89 µmol/L. The imaging showed gradual enhancement and visualization of both the renal main trunk arteries and segmental arteries. The renal cortex enhanced rapidly and homogeneously, with the medulla showing progressive enhancement from the periphery to the center (Figures 3A,C). The TIC curve displayed a near-normal rise; however, the falling phase exhibited an almost “platform-shaped” appearance, indicative of slow excretion (Figures 3B,D). Quantitative parameters were presented at Table 2.

**Table 2:** Quantitative parameters of CEUS in the second patient.

Midray Resonna R9	Date	TTP	RT	PI	AS	DS	AUC
Right	2d	18.00	9.90	45.78	4.32	0.14	3,253.81
Left	2d	20.70	10.90	46.46	3.99	0.13	3,327.90

CEUS, contrast-enhanced ultrasound; TTP, peak time in seconds; RT, rise time in seconds; PI, peak intensity in dB; AS, ascending slope; DS, descending slope; AUC, area under the curve.

## Discussion

CsA serves as a crucial immunosuppressive agent in preventing graft rejection following various allogeneic transplantation procedures, including renal, hepatic, cardiac, and hematopoietic stem cell transplantation. However, its acute renal toxicity is primarily attributed to its ability to constrict arterioles, resulting in reduced renal blood flow (4). This toxicity results in a rapid decline in glomerular filtration function, manifesting as oliguria, elevated blood urea nitrogen, and increased creatinine levels (5). This functional renal toxicity is often dose-dependent and generally does not lead to permanent kidney damage. The effects of CsA can be reversed by reducing the dosage or discontinuing its use (6).

The 1-year-and-11-month-old child was definitively diagnosed with AKI, although the underlying cause remained unclear. Owing to the absence of shock manifestations, low cardiac output as a contributor to renal perfusion was excluded. Although the human terminal complement complex C5b-9 levels were slightly elevated at 261 (reference range: 72–252) ng/ml, suggesting potential abnormal complement activation that could lead to thrombotic microangiopathy (TMA), multiple peripheral blood smear examinations did not reveal any fragmented red blood cells, indicating no evidence of hemolysis associated with TMA. Additionally, ADAMTS13 activity monitoring showed no reduction, and the patient's later-stage treatment outcomes were inconsistent with those typically observed in TMA.

The patient received numerous drugs before and after the transplantation, raising concerns about drug-induced renal injury. Cyclophosphamide (CTX), CsA, and vancomycin were identified as possible contributors. The cumulative dose of CTX was 250 mg/kg, administered in two doses, with moderate dosage and without therapeutic drug monitoring. CTX causes functional disturbances in both the glomerulus and tubule, in a dose-dependent manner. Manifestations include glomerular or tubular proteinuria, decreased glomerular filtration rate (GFR; reversible), and diminished renal concentrating function (7, 8). However, as the patient did not receive large doses of CTX and exhibited no proteinuria, AKI attributed to CTX was considered unlikely. The trough concentration of vancomycin was slightly elevated at 24.9 (reference range: 10–20) mg/L. Vancomycin is primarily filtered by the glomerulus and cleared via proximal tubular reabsorption and secretion. Its nephrotoxicity is attributed to its accumulation, leading to necrosis of proximal renal tubular cells (9). This mechanism, however, does not align with the poor renal cortical perfusion observed in the patient's kidney CEUS. It is hypothesized that the patient's AKI impaired vancomycin excretion, resulting in its accumulation within the body and an elevated blood drug concentration.

Before AKI, the trough concentration of CsA ranged from 66.4 to 134.1 µg/L. However, after the onset of AKI, the trough concentration increased considerably to 332.6 µg/L. According to a pediatric research report, a CsA trough level exceeding 200 µg/L is a predictor of acute AKI (10). Furthermore, a study by Kennedy et al. demonstrated that a CsA trough level greater than 250 µg/L is associated with a 100% incidence rate of AKI (11). Several studies have shown that CsA toxicity leads to constriction of the afferent arteriole as well as endothelial cell swelling and degeneration, resulting in decreased renal blood flow and GFR, ultimately manifesting as oliguria, elevated blood pressure, and AKI (1, 12).

This patient exhibited persistent oliguria, high blood pressure, significantly elevated creatinine levels, and poor renal cortical perfusion—symptoms consistent with CsA toxicity. This suggests that the patient's AKI was likely related to CsA toxicity. After discontinuing CsA and initiating continuous renal replacement therapy, a follow-up kidney CEUS showed that the TIC curve resumed its normal rising-decreasing pattern. Bilateral kidney perfusion improved compared to previous observations, with more noticeable improvement in the left kidney, indicating recovery of the glomerular capillary network. This also indirectly confirmed that the AKI was caused by CsA toxicity. Following the suspension of CsA, the patient's urine output, creatinine levels, and blood pressure gradually returned to normal.

Ibuprofen is a nonsteroidal anti-inflammatory drug that inhibits the production of prostaglandins and their metabolites. This inhibition can lead to significant vasoconstriction in the glomerular afferent arterioles, consequently reducing renal cortical perfusion (13, 14). Ibuprofen can also induce acute interstitial nephritis through lymphocyte activation, primarily via an immunological mechanism (15). This condition is characterized by acute renal dysfunction with or without oliguria and is often accompanied by fatigue, fever, joint pain, and other nonspecific symptoms (16). Renal tubular dysfunction may result in urine with low specific gravity and osmolarity; tubular proteinuria; and disturbances in water, electrolyte, and acid-base balance.

The other 14-year-old child developed AKI after ingesting a high dose of ibuprofen over a short time, accompanied by symptoms such as abdominal pain, nausea, fatigue, and hypersomnia. Laboratory tests revealed elevated levels of serum cystatin C, NGAL, creatinine, and other markers, indicating a decreased GFR and subsequent AKI. Unfortunately, a kidney CEUS was not promptly performed but performed after improvement in urine output and renal function. The imaging showed rapid and uniform enhancement of the renal cortex, with the TIC rapidly rising and slowly descending. The descending phase of the curve plateaued, suggesting that although both kidney cortices were well-perfused with blood, cortical blood washout and excretion were significantly delayed.

The impact of ibuprofen poisoning on renal blood flow can be summarized as follows: (1) increased resistance in the afferent arterioles leads to reduced perfusion in the renal cortex; (2) injury occurs to the tubules around the interstitium. At the time of writing, the patient had normal urine output, along with normal serum creatinine and cystatin C levels, suggesting an unobstructed urinary tract from the glomerulus, Bowman's capsule, and tubules to the collecting ducts, ureters, and the bladder. Kidney CEUS showed adequate cortical perfusion, indicating unobstructed blood flow from the descending aorta to the glomerulus. Based on this, it was hypothesized that the delay in cortical excretion observed on CEUS was related to delayed blood flow in the peritubular capillary network. In addition, the patient's bilateral kidney color Doppler ultrasound demonstrated continuous flow in the interlobular veins, ruling out vascular obstruction as a cause of posterior nephropathy.

Considering the patient's medical history, it was believed that ibuprofen poisoning caused inflammation and edema around the renal cortex and tubules, potentially leading to blood flow obstruction around the tubules. This suggests that during the recovery period of AKI, even when the urinary tract appears unobstructed and the urine output and serum creatinine have improved, there may still be hidden damage to the capillaries around the tubules. Moreover, the recovery of blood perfusion in the small vessels around the tubules may be delayed compared to the restoration of perfusion in the glomerulus. Failing to recognize this potential capillary damage may delay the diagnosis and treatment of “hidden” AKI. To prevent exacerbating kidney injury, further drug-induced renal damage, hemodynamic fluctuations, and excessive inflammatory reactions should be avoided.

Currently, there is a paucity of studies exploring the significance of the rising and falling branches of renal CEUS quantitative analysis curves in relation to the anatomical locations of damage within the renal microcirculation. This study aims to analyze, for the first time, the clinical features and renal CEUS images of two children affected by drug overdose or intentional and proposes the following: (1) in addition to being influenced by perfusion of the large circulation, the structure and function of the glomerulus itself—comprising the afferent artery, glomerular capillary network, and efferent artery—significantly influence the perfusion rate and intensity of the rising branch of the curve; (2) the descending branch of the curve is affected not only by renal vein return but also by factors related to the capillaries surrounding the renal tubules. By exploring these anatomical and functional relationships, this study aims to enhance the understanding of blood flow pathways within renal microcirculation, thereby contributing to a deeper knowledge of renal perfusion dynamics.

Intravenous CEUS proves valuable in pediatric AKI diagnosis, enabling real-time, repeatable assessment of renal microcirculation without radiation risks and no renal toxicity. The technique facilitates anatomical localization of microcirculatory abnormalities, aiding in understanding AKI pathophysiology while optimizing hemodynamic monitoring in children.

Several study limitations should be noted. First, the optional inclusion of drug-induced AKI cases resulted in a relatively homogeneous disease spectrum. Second, the small sample size limited comprehensive data analysis. Finally, the absence of normal controls may affect result reliability. While larger cohorts are needed to fully establish pediatric renal CEUS dataset, this remains an important focus for future research.

## Data Availability

The original contributions presented in the study are included in the article/Supplementary Material, further inquiries can be directed to the corresponding authors.
